# Modulating Exciton Dynamics Through Fluorescent Side Group Incorporation in Benzodithiophene-Benzotriazole-Isoindigo Terpolymers

**DOI:** 10.3390/polym18121554

**Published:** 2026-06-22

**Authors:** René Hauyón, Yasmín Pérez, Daniela Zúñiga, Scarlet Araya, Bastian Camacho, Pablo Thomas, Cesar Saldías, Denis Fuentealba, Claudio A. Terraza, Felipe A. Angel, Ignacio A. Jessop

**Affiliations:** 1 Facultad de Química y de Farmacia, Pontificia Universidad Católica de Chile, Santiago 7820436, Chile; rahauyon@uc.cl (R.H.); casaldia@uc.cl (C.S.); dlfuente@uc.cl (D.F.); cterraza@uc.cl (C.A.T.); faangel@uc.cl (F.A.A.); 2 Organic and Polymeric Materials Research Laboratory, Facultad de Ciencias, Universidad de Tarapacá, Arica 1000007, Chilez.candia.daniela@gmail.com (D.Z.); b.camachoromo@gmail.com (B.C.);; 3 Centro de Energía, CE-UC, Pontificia Universidad Católica de Chile, Santiago 7820436, Chile; 4 Centro de Nanotecnología y Materiales Avanzados, CIEN-UC, Pontificia Universidad Católica de Chile, Santiago 7820436, Chile

**Keywords:** π-conjugated terpolymers, fluorescent side groups, intramolecular FRET, exciton dynamics

## Abstract

In this work, we investigated the incorporation of a fluorescent side group, fluorescein octyl ester (FOE), in benzodithiophene-based donor–acceptor terpolymers as a strategy to modulate excited-state behavior. Three FOE-containing terpolymers (**P2-iIa-c**), obtained at different polymerization times, were systematically evaluated against an analogous material without the fluorescent pendant unit (**P1-iI**). Thermal analysis revealed good thermal stability and an increase in glass transition temperature upon FOE incorporation, suggesting restricted segmental mobility and increased conformational constraints within the conjugated backbone. Optical characterization showed distinct absorption spectra with reaction time and shorter fluorescence lifetimes for the FOE-containing materials, consistent with the presence of additional excited-state deactivation pathways and intramolecular energy transfer processes within the terpolymer backbone. An approximate estimation of energy transfer efficiencies (≈60–65%) suggested that such processes may be operative within the system. Cyclic voltammetry measurements showed only minor variations in HOMO and LUMO energy levels between **P1-iI** and **P2-iIa-c** series, indicating that the conjugated backbone predominantly determined the frontier orbital energies despite side chain modification. Furthermore, photocurrent measurements from the bilayer device configuration exhibited a systematic increase in photocurrent for the FOE-containing material, supporting the role of excitonic modulation, rather than significant changes in interfacial energetic alignment. These results suggest that fluorescent side chain incorporation provides an effective strategy for regulating exciton dynamics while maintaining the electronic structure of the donor–acceptor terpolymer.

## 1. Introduction

Over the past few decades, solar photovoltaic (PV) technologies have attracted considerable attention as a promising solution to address global energy challenges and reduce reliance on fossil fuels [1,2,3,4,5,6]. Although silicon-based PV solutions still dominate the solar market, conjugated polymer-based solar cells (PSCs) have received significant attention as a promising alternative, owing to the advantages of transparency, light weight, compatibility with low-cost solution processing, and mechanical flexibility in contrast to silicon, perovskite, and other inorganic PV-based materials [7,8,9,10]. Recent developments in donor and, particularly, in acceptor materials, as well as improvements in device fabrication and morphological optimization, have driven bulk-heterojunction (BHJ) PSCs to reach power conversion efficiencies (PCEs) exceeding 20% [11,12,13,14,15,16,17]. Despite these impressive performances, further improvements in efficiency, operational, and long-term morphological stability remain critical challenges towards commercialization, in addition to understanding and controlling the photogeneration processes [18,19,20,21].

Significant efforts have been devoted to the design of high-performance conjugated polymer donors with good solubility in common organic solvents, wide and strong absorption from the visible to near-infrared (NIR) region, suitable energy levels that align with the acceptor materials, high electron mobility, and appropriate morphology to ensure compatibility in blend systems [8,9,22,23,24,25]. These polymers take advantage of the “push–pull” effect between adjacent electron-donating (D) and electron-withdrawing (A) monomers, where rational choice of the D and A units is key in the tuning of their optical and electrochemical properties [9,10,11]. Among them, D-A polymers based on a benzo[1,2-*b*:4,5-*b*’]dithiophene (DBT) core have gained particular attention due to their strong absorption, high planarity, and good self-organization properties, reaching record power conversion efficiencies [26,27,28].

Beyond conventional D-A copolymers, random terpolymers incorporating a third monomer along the conjugated backbone have emerged as an effective strategy to optimize optical, electrochemical, photovoltaic, and morphological properties. As reported in the literature, the introduction of a third monomer has been employed to optimize absorption profiles or adjust energetic alignment in PSCs [25,29,30,31,32,33,34]. In parallel, ternary PSC systems have been reported as an interesting alternative to improve device efficiency and stability simultaneously. By incorporating a third component into a binary system, the light-absorption bandwidth of the active layer is expanded. Simultaneously, photophysical effects such as cascading energetics and Förster resonance energy transfer (FRET) are enhanced, and the blend morphology can be adjusted, thereby improving overall device performance [1,35,36,37,38,39,40].

While most molecular strategies in conjugated polymers have traditionally focused on modifying the backbone to tune electronic energy levels, less attention has been given to controlling how excitons behave once generated. However, exciton migration, dissociation, and decay are central in determining the photovoltaic performance of these materials. From these perspectives, designing innovative donor materials is essential to harness the unique advantages of terpolymers and ternary solar cell systems, while accounting for exciton dynamics. In this regard, we focused on exploring strategies to integrate characteristics of these systems to improve the performance and efficiency of PSCs. The incorporation of fluorescent side groups capable of acting as intramolecular energy donors offers an alternative strategy for terpolymers [25]. By promoting FRET-type energy transfer toward the conjugated backbone, these pendant units or third components in ternary solar cells can modulate exciton dynamics and improve light harvesting processes while maintaining the electronic structure largely unaffected [41,42,43,44,45,46].

In a previous study [25], we reported a π-conjugated terpolymer based on a benzodithiophene derivative, 4,8-bis(2-ethylhexyloxy)benzo[1,2-*b*:4,5-*b*’]dithiophene (1D-BDT), and 2-hexyl-4,7-di(2-thienyl)-2*H*-benzo[*d*][1,2,3]triazole (BTzC6) functionalized with a fluorescein octyl ester (FOE) attached to the C6 carbon atom of the side chain. This terpolymer demonstrated that intramolecular FRET can occur from the FOE side group to the conjugated backbone. However, the influence of FOE incorporation and the FRET effect on photocurrent generation remained inconclusive.

**Scheme 1:**
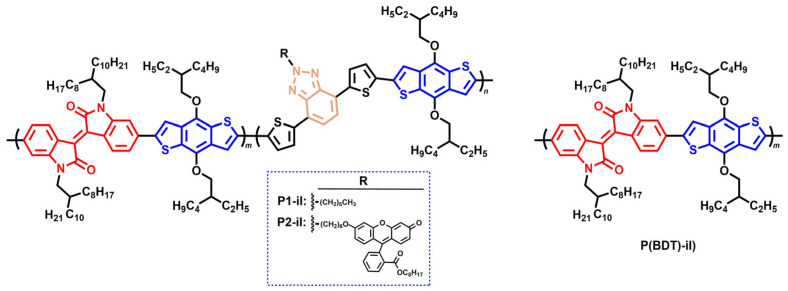
A schematic representation of the 1D-BDT (blue), BTz (orange), and iI (red)-based terpolymers and copolymer **P(BDT-iI)**.

Extending this work, herein we synthesized two donor–acceptor π-conjugated terpolymers (**P1-iI** and **P2-iIa-c**) to systematically investigate the structural and photophysical impact of the FOE unit. Both materials have 1D-BDT moieties as the electron donor unit, combined with a BTzC6 core and *N,N’*-bis(2-octyldodecyl)isoindigo (iI) as weak and strong electron-accepting building blocks, respectively, to extend absorption into the NIR region, improve light-harvesting, and reduce the optical bandgap [47,48,49,50,51]. **P1-iI** had a hexyl side chain attached to the central nitrogen atom of the BTz core (BTzC6), while P2-iI incorporated a FOE unit via a hexyl spacer on the same position (BTzC6FOE) (Scheme 1). Through systematic thermal, photophysical, electrochemical, and photovoltaic analyses, we evaluated the influence of introducing FOE along the terpolymeric backbone on excited-state behavior and overall material properties. Particular emphasis was placed on determining whether the incorporation of FOE can modulate exciton dynamics through intramolecular energy transfer processes while minimally perturbing the frontier orbital energies of the terpolymeric backbones.

## 2. Materials and Methods

### 2.1. Materials

All chemical reagents and solvents, including 2,6-bis(trimethyltin)-4,8-bis-ethylhexyloxy-benzo[1,2-*b*:4,5-*b*’]dithiophene (1D-BDT), were purchased from Sigma-Aldrich (St. Louis, MO, USA) and Merck (Rahway, NJ, USA) and were used directly without any further purification unless otherwise noted. *N*-Bromosuccinimide (NBS) was recrystallized twice from hot water. The polymerization solvents were degassed and stored over activated 3 Å molecular sieves under an inert atmosphere, while the other solvents were ACS grade. Monomers 2-(6-hexyl)-4,7-bis(5-bromothiophen-2-yl)-2*H*-benzo[*d*][1,2,3]triazole (BTzC6), octyl 2-(3-(6-(4,7-bis(5-bromothiophen-2-yl)-2*H*-benzo[*d*][1,2,3]triazol-2-yl)hexyloxy)-6-oxo-6*H*-xanthen-9-yl)benzoate (BTzC6FOE), and 6,6′-dibromodi(*N,N’*-2-octyldodecyl)isoindigo (iI) were synthesized according to previous reports [25,52]. Their structures were confirmed by ^1^H and ^13^C NMR spectroscopy (see Supplementary Materials).

### 2.2. Measurements

^1^H and ^13^C NMR spectra were recorded on a Bruker AVANCE III HD 400 spectrometer in deuterated solvents. Chemical shifts were reported as δ values (ppm) relative to an internal tetramethylsilane (TMS) standard. Number-average (*M_n_*) and weight-average (*M_w_*) molecular weights were determined by size exclusion chromatography (SEC) at 25 °C with a high-pressure liquid chromatography (HPLC) Knauer pump, three PLgel 5µMixed-C columns, and a static light-scattering (EA-02 Dawn Eos Enhanced Optical System, Artisan TG, Champaign, IL, USA) detector. The flow rate was 1.0 mL·min^−1^ using tetrahydrofuran (THF) as eluent. All samples were prepared at 1.0 mg·mL^−1^ in THF and were filtered through a 0.45 µm nylon filter. The calibration curve was made with a series of monodisperse polystyrene standards. Thermogravimetric analysis (TGA) measurements were performed with a PerkinElmer Termogravimetric Analyzer TGA 4000 (PerkinElmer Shelton, CT, USA) from 25 °C to 800 °C at a heating rate of 20 °C·min^−1^. Differential Scanning Calorimetry (DSC) analysis of samples was performed in a PerkinElmer DSC 4000 from 25 to 300 °C at 20 °C·min^−1^ heating and cooling rates. Both TGA and DSC measurements were carried out under a N_2_ atmosphere with a flow of 20 mL·min^−1^. UV-vis absorption spectra were collected in a Shimadzu UV-1900i spectrophotometer (Shimadzu, Kyoto, Japan) using 1 cm path length quartz cells. Fluorescence spectra were recorded with a FS5 spectrofluorometer using a 1.0 nm width slit for emission. The samples were excited at the polymer’s maximum absorption wavelength in solutions diluted to 0.1 a.u. to minimize self-absorption. Photoluminescence lifetime measurements in solution were carried out using the time-correlated single photon counting (TCSPC) method in a LifeSpecII fluorescence life-time spectrometer equipped with an Edinburgh Instruments picosecond pulsed diode laser, model EPL-375 (Edinburgh Instruments, Livingston, UK) (wavelength = 375.6 nm, pulse width = 61.1, 175 ps, average power = 5 mW). The decays were fitted with the use of an IRF and using the deconvolution fit methodology. Cyclic voltammetry (CV) experiments were conducted on an OrigaFlex potentiostat (OrigaLys, Rillieux-la-Pape, France) at a scan rate of 100 mV·s^−1^ using a glassy carbon working electrode, Ag/AgCl as reference electrode, and Pt wire as counter electrode in an anhydrous and argon-saturated solution of 0.01 M of tetrabutylammoniumhexafluorophosphate (Bu_4_NPF_6_) in acetonitrile. A flow of high purity argon was maintained during the measurements. The working electrode was modified by drop-casting 2 mg·mL^−1^ solutions of polymers in chloroform. FOE was measured in a 1 mM solution containing 0.01 M Bu_4_NPF_6_ in acetonitrile. Under these experimental conditions, the oxidation potential (*E^ox^*) of ferrocene was 0.426 V versus Ag/AgCl. The HOMO and LUMO energy levels were determined from the oxidation and reduction onset potentials in the voltammogram profiles.

### 2.3. PSC Device Fabrication

For device fabrication, 1.5 × 1.5-inch glass substrates with a 1000 Å pre-patterned indium tin oxide (ITO) layer were used. The substrates showed a surface resistance of ~15 Ω·sq^−1^ and ~90% optical transparency across the solar spectrum. The substrates were cleaned in a laminar flow hood, including scrubbing with detergent solution, rinsing with deionized water, and ultrasonic baths for 10 min in DI water, acetone, and isopropanol. Lastly, nitrogen gas was used to dry the substrates. All layers based on small molecules were prepared by vapor deposition in a vacuum chamber (<5.0·10^−6^ Torr). The deposition of layers onto the substrates began with an 8 nm molybdenum oxide (MoO_x_) layer at approximately 0.3 Å·s^−1^ on the ITO substrate, serving as the hole-injecting layer (HIL). Subsequently, 300 µL of a chloroform solution of **P1-iI** and **P2-iIa-c**, with concentrations ranging from 1.0 to 3.0 mg·mL^−1^, was spin-coated onto the ITO|MoOx substrates at 1000 r.p.m. for 45 s, yielding films with thicknesses between 15 and 49.5 nm, as measured using a Profilm3D optical profilometer (KLA Corporation, Milpitas, CA, USA). Prior to deposition, all solutions were filtered through a PTFE 0.22 µm syringe filter. To complete device fabrication, a 40 nm layer of fullerene C_60_ was deposited at a rate of 4.0 Å·s^−1^ as the acceptor layer following a bilayer configuration, while an 8 nm layer of 4,7-diphenyl-1,10-phenanthroline (Bphen) was deposited as the electron transport layer (ETL), followed by a 1 nm layer of 8-hydroxyquinolinato lithium (Liq) as the electron-injecting layer (EIL). The deposition rates for Bphen and Liq were 4.0 Å·s^−1^ and 1.0 Å·s^−1^, respectively. Finally, a 100 nm top aluminum electrode was deposited at a constant rate of 5.0 Å·s^−1^. Four identical devices with an active area of 0.1 cm^2^ were produced on a single substrate, with the device architecture ITO|MoO_x_|**P1-iI**/**P2-iIa-c**|C_60_|Bphen|Liq|Al.

The current density–voltage (J-V) characteristics were recorded with a Keithley 2400 (Keithley Instruments, Solon, OH, USA) source meter in the dark and under irradiation at 80 mW·cm^−2^ generated by a Solux 3SS4736 with a 50W 47K halogen lamp (EiKO Global, LLC, Lenexa, KS, USA). The intensity was calibrated with a Hamamatsu S1787-12 silicon photodiode. External quantum efficiency (EQE) measurements were performed with a SpectraPro 275 monochromator (Artisan TG, Champaign, IL, USA).

### 2.4. General Procedure for Polymerizations

In a 25 mL oven-dry two-neck round-bottom flask equipped with a magnetic stirring bar, monomers were charged (0.15 mmol of 1D-BDT, 0.075 mmol of BTzC6 or BTzC6FOE, and 0.075 mmol of iI) for the synthesis of **P1-iI** and **P2-iIa-c**. For the reference copolymer **P(BDT-iI)**, equimolar amounts of BDT (0.15 mmol) and iI (0.15 mmol) were used. Pd_2_(dba)_3_ (2 mol%) and P(*o*-tol)_3_ (8 mol%) were then added to the flask. The flask was connected to a condenser, sealed with septa, and subjected to three vacuum/nitrogen cycles. Anhydrous, oxygen-free toluene (30 mL) was then added via a syringe. The reaction mixture was stirred under nitrogen and heated to 110 °C using an oil bath for the specific reaction time. For **P1-iI**, the reaction time was 30 min. The FOE-containing terpolymers P2-iIa-c were synthesized using different reaction times (25 min, 50 min, and 3.5 h for P2-iIa, P2-iIb, and P2-iIc, respectively) to evaluate how polymerization time influences the evolution of the polymer structure and its resulting physicochemical properties. The reaction time for the copolymer **p(BDT-iI)** was 30 min. After completion of the reaction, the mixture was removed from the oil bath, and *o*-dichlorobenzene (5 mL) was added. After cooling at room temperature, the crude polymer was precipitated into methanol and collected by filtration through a 0.45 µm nylon membrane. The solid was then washed by successive Soxhlet extraction with acetone, *n*-hexane, and chloroform to remove unreacted monomers, undesired by-products, and low-molecular-weight fractions. The chloroform fraction was concentrated to 5–10 mL and slowly poured into methanol. The resulting black solids were filtered through a 0.45 µm nylon membrane and dried at 50 °C for 24 h. **P1-iI** was obtained in 23% yield. FOE-containing terpolymers **P2-iIa**, **P2-iIb**, and **P2-iIc** were obtained in 37%, 43%, and 50% yields, respectively, while **P(BDT-iI)** was isolated in 90% yield. All polymers were soluble in common solvents such as chloroform, chlorobenzene, *o*-dichlorobenzene, among others. Synthetic route, monomer characterization, and polymer ^1^H NMR spectra are provided in the Supplementary Materials.

## 3. Results and Discussion

### 3.1. Synthesis of Polymers P1 and P2

The synthetic strategy for obtaining **P1-iI** and **P2-iIa-c** series was based on a Stille polycondensation approach using commercially available 1D-BDT derivative and iI and BTz derivatives described in the literature [25,53]. Our group has previously reported the synthesis of the FOE-functionalized monomer BTzFOE [25]; therefore, details of its preparation were not discussed here. Characterization data are provided in the Supporting Information.

**P1-iI** and **P(BDT-iI)** were synthesized under identical Stille coupling conditions to allow for a direct comparison of their structural and physicochemical properties. In our previous work [25], the optical and electrochemical contributions of the BDT-BTzC6FOE segment were exhaustively characterized. Thus, the synthesis of **P(BDT-iI)** aimed to isolate and identify the specific influence of the BDT-iI segment within the terpolymeric backbone. Due to differences in composition and degree of polymerization, this material was used only as a qualitative reference, allowing for differentiation between the effects of the BTz and iI-based monomers. For the FOE-containing terpolymers (**P2-iIa-c**), polymerizations were carried out at three different reaction times to evaluate the influence of reaction time on the evolution of the polymer structure and its resulting physicochemical properties. Although the FOE units are electronically decoupled from the conjugated backbone, their incorporation may influence the polymerization process through steric and conformational effects. In particular, the bulky and planar aromatic structure of the FOE side group may hinder the approach and coupling efficiency of reactive sites during Stille polycondensation [54,55]. In addition, possible intermolecular interactions involving FOE moiety could further influence solubility and the effective reactivity of growing chains.

The molecular weights and dispersity values were determined by SEC, and the results are summarized in Table 1. **P2-iIa-c** series exhibited number-average molecular weights (*M_n_*) in the range of 13.9–15.7 kDa, corresponding to relatively low degrees of polymerization (DP ≈ 6). In contrast, terpolymer **P1-iI** reached a significantly higher *M_n_* (48.3 kDa), corresponding to a DP of approximately 24. The relatively similar *M_n_* values observed for the **P2-iIa-c** series at different reaction times suggest that polymer chain growth predominantly occurs during early stages rather of the reaction, with limited further increase upon prolonged polymerization [56,57].

The *M_n_* value of **P1-iI** terpolymer was also higher than that of **P(BDT-iI)**, despite both containing BDT-iI segments, which could be attributed to the pre-aggregation tendency reported for isoindigo-based systems [49,58,59]. However, this effect does not appear to limit chain growth in **P1-iI**, suggesting that the incorporation of BDT-BTzC6 segments improves solubility and facilitates chain propagation during polymerization.

In the case of **P2-iIa-c** series, the incorporation of the bulky FOE units through the BTz moiety may induce steric hindrance around the reactive sites, which may reduce the efficiency of Stille coupling during polymerization and lead to shorter polymer chains [54,55]. Although FOE incorporation could be expected to enhance solubility due to its aromatic and nonpolar nature, the relatively low molecular weights suggest that steric factors dominate over solubility effects in determining the final chain length. Additionally, the possibility of intermolecular interactions involving the aromatic FOE units, such as π-π stacking, cannot be excluded and may contribute to aggregation observed experimentally.

**Figure 1:**
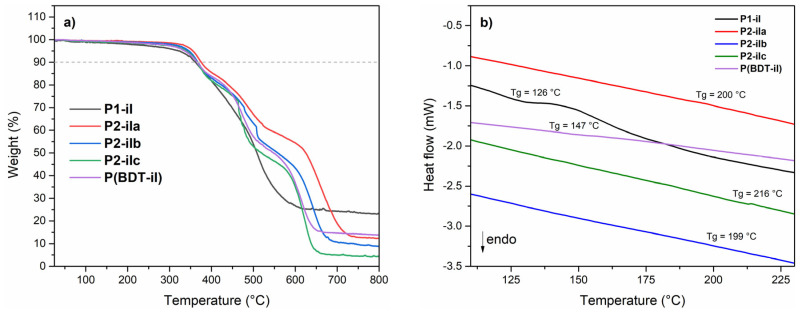
(**a**) TGA curves and (**b**) DSC traces of terpolymers **P1-iI** and **P2-iIa-c** and copolymer **P(BDT-iI)**.

Thermal stability of the polymers was assessed by TGA, where the onset decomposition temperatures corresponding to 10% weight loss (TDT_10%_) ranged from 362 °C to 376 °C (Table 1 and Figure 1), indicating good thermal stability. The small difference observed between these values (Δ = 14 °C) suggests that the incorporation of BTz moieties bearing C6 side chains or their arylated analogs with FOE does not significantly affect the thermal stability of the materials. In addition, all materials exhibited a multistep degradation profile, with a main decomposition stage occurring between 350 and 550 °C, which can be associated with the degradation of the conjugated polymer backbone. Differences in residual mass at higher temperatures suggest variations in char formation and structural organization among polymers. The glass transition temperatures (*T_g_*), determined by DSC, were significantly higher for **P2-iIa-c** terpolymers (199–216 °C) than for **P1-iI** (126 °C) and **P(BDT-iI)** (147 °C). No clear crystallization or melting transitions were observed within the temperature range studied. Instead, the *T_g_* transitions were relatively broad and not sharply defined, which can be attributed to the restricted cooperative molecular rearrangements imposed by the rigid aromatic conjugated backbones. The higher *T_g_* values observed for the **P2-iIa-c** series may reflect reduced segmental mobility associated with the presence of bulky FOE side groups and/or enhanced interchain interactions. Overall, these results indicate that FOE incorporation enhances backbone rigidity without compromising thermal stability.

### 3.2. Optical and Electrochemical Characterization

**Figure 2:**
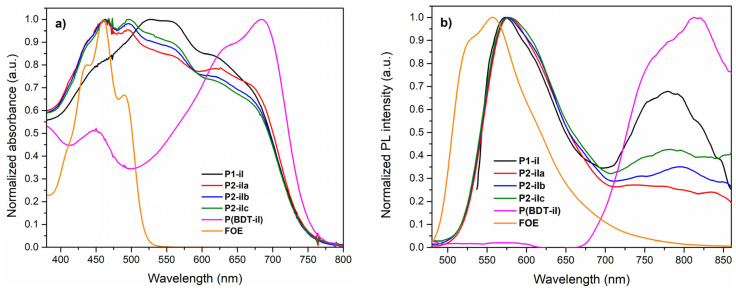
(**a**) Normalized Uv-vis and (**b**) fluorescence spectra of terpolymers **P1-iI** and **P2-iIa-c**, the copolymer **P(BDT-iI)** and the FOE antenna in dilute CHCl_3_ solutions.

The UV-vis absorption spectra of the terpolymers **P1-iI** and **P2-iIa-c**, the copolymer **P(BDT-iI)** and the FOE antenna in chloroform dilute solutions are shown in Figure 2, and the corresponding data are summarized in Table 2. **P1-iI** exhibited a broad absorption band with a maximum at 527 nm, together with two additional bands around 560 nm and 620 nm, which are attributed to intramolecular charge transfer (ICT) transitions between donor and acceptor units (BDT-BTzC6 and BDT-iI, respectively). Consistent with our previous findings [25], in the absence of FOE, the terpolymer showed a red-shifted absorption compared to those with FOE, which can be attributed to its higher degree of polymerization and extended effective conjugation length. The copolymer **P(BDT-iI)** showed an even more pronounced red-shift (λ_max_ = 684 nm, with a shoulder around 630 nm), despite its lower number-average molecular weight. This could suggest that factors beyond molecular weight, such as backbone composition and connectivity, also play a role in determining the effective conjugation length. In particular, the absence of the BTz unit in **P(BDT-iI)** may favor a more efficient conjugation between BDT and iI units, whereas in terpolymers, the incorporation of BTz-based segments could introduce slight disruptions in conjugation along the backbone. At the same time, in the **P1-iI** and **P2-iIa-c** systems, the random incorporation of three monomers with different reactivities, together with the limited degree of polymerization observed for the FOE-containing series, is expected to generate a variety of local segment arrangements and conjugation pathways along the backbone. As a result, variations in backbone connectivity and effective conjugation length are expected. Therefore, the observed spectral differences likely arise from a combined effect of segmental distribution, backbone connectivity, and effective conjugation length within the terpolymeric chains.

The **P2-iIa-c** terpolymers also exhibited a broad absorption band in the visible region. As the reaction time increases from 25 min to 3.5 h, the absorption maximum at 463 nm, related to FOE absorption, remains essentially unchanged, while the bands at 496 nm and 550 nm become more prominent. These latter bands were assigned to BDT-BTzC6FOE segments along the polymer backbones. In contrast, the band at 620 nm, associated with BDT-iI segments, gradually decreases with increasing reaction time. These spectral changes are consistent with differences in the relative contribution of BTzC6FOE and iI monomers to 1D-BDT monomer during polymer growth. As a result, the relative contribution of BDT-BTzC6FOE appears to increase with reaction time, as inferred from the evolution of the absorption spectra. Notice that this interpretation should be regarded as indirect evidence of compositional variation rather than a direct determination of segmental distribution. Considering that the molecular weights of the **P2-iIa-c** series remain essentially constant, these spectral changes are more likely associated with variations in segment distribution rather than differences in chain length.

The fluorescence spectra of the terpolymers, copolymer, and FOE in dilute chloroform solutions are presented in Figure 2, and their photophysical parameters are summarized in Table 2. All fluorescence spectra were normalized to their maximum intensity to facilitate comparison of spectral features; therefore, differences in absolute emission intensity between materials cannot be directly assessed from these spectra. After excitation at their respective absorption maxima, emission bands centered between 567 nm and 574 nm were observed in terpolymers **P1-iI** and **P2-iIa-c**, which were attributed to the BDT-BTzC6FOE segments [25]. In addition, secondary emission bands were observed in the 780–795 nm range and were attributed to the BDT-iI segments, as supported by the emission spectra of the **P(BDT-iI)** copolymer. Isoindigo-based materials tend to exhibit intrinsically low fluorescence quantum yields due to efficient nonradiative decay from the S1 excited state, often associated with photoinduced electron transfer processes [49]. As a result, emission from BDT-iI segments is expected to be less intense compared to higher-energy segments. The emission spectrum of FOE exhibited extensive overlap with the absorption bands of the terpolymers, especially those associated with BDT-BTzC6FOE segments. This spectral overlap provides favorable conditions for intramolecular energy transfer processes (FRET-like) from FOE to the conjugated backbone.

Interestingly, although the UV-vis spectra showed an increasing contribution of BDT-BTzC6FOE segment with increasing reaction time, the fluorescence spectra reveal an increase in the relative contribution of the BDT-iI emission band with respect to the main emission peak. This apparent discrepancy suggests that the emission response is not solely governed by segmental composition but also by exciton migration and redistribution along the terpolymer backbone. A plausible mechanism involves a cascade energy transfer process, in which excitons initially generated in FOE units may be transferred to BDT-BTzC6FOE segments and subsequently redistributed via intrachain migration to lower-energy BDT-iI segments, which can act as preferential energy sinks. Because these segments exhibited intrinsically low radiative efficiency, their contribution to the overall emission is expected to be limited. In this context, FOE incorporation does not appear to enhance emission intensity but instead modifies exciton dynamics within the terpolymer. This behavior may involve additional non-radiative pathways or redistribution processes that compete with radiative recombination, resulting in changes in the relative emission profile compared to the **P1-iI** terpolymer.

Time-resolved fluorescence measurements provide further insight into the excited-state dynamics within the terpolymers. As shown in Table 3, the FOE antenna exhibits a monoexponential decay with a lifetime of 2.64 ns [25]. In contrast, the **P2-iIa-c** terpolymers displayed shorter dominant lifetime components (τ_1_ ≈ 0.9–1.0 ns), together with secondary longer-lived components (τ_2_ ≈ 2–3 ns). This large decrease in the excited-state lifetime compared with FOE suggests the presence of additional deactivation pathways in the terpolymer system. Considering the significant spectral overlap between FOE emission and terpolymer absorption, together with the steady-state fluorescence results, this behavior is consistent with the occurrence of intramolecular energy transfer processes from the FOE units to the conjugated backbone. An approximate estimation of energy transfer efficiency based on the observed lifetimes yields in the range of 60–65%, estimated according to ηET = 1 − τDA/τD, supporting efficient excited-state coupling between FOE and the conjugated backbones. The presence of two decay components also suggests conformational or compositional heterogeneity within the terpolymer structure, leading to regions with different local environments and energy transfer efficiencies.

Although structural and photophysical differences among terpolymers **P1-iI** and **P2-iIa-c** were observed, their optical bandgaps were similar (*E_g_^opt^* ≈1.65–1.66 eV). This indicates that the electronic structure of the conjugated backbone is preserved with the incorporation of the FOE antenna and variations in segment distribution. The copolymer **P(BDT-iI)** exhibited a slightly lower bandgap (1.63 eV), consistent with a more extended conjugation and red-shifted absorption and emission. These results suggest that while FOE incorporation influences exciton dynamics and energy redistribution processes, it does not alter the optical bandgap of the backbone.

**Figure 3:**
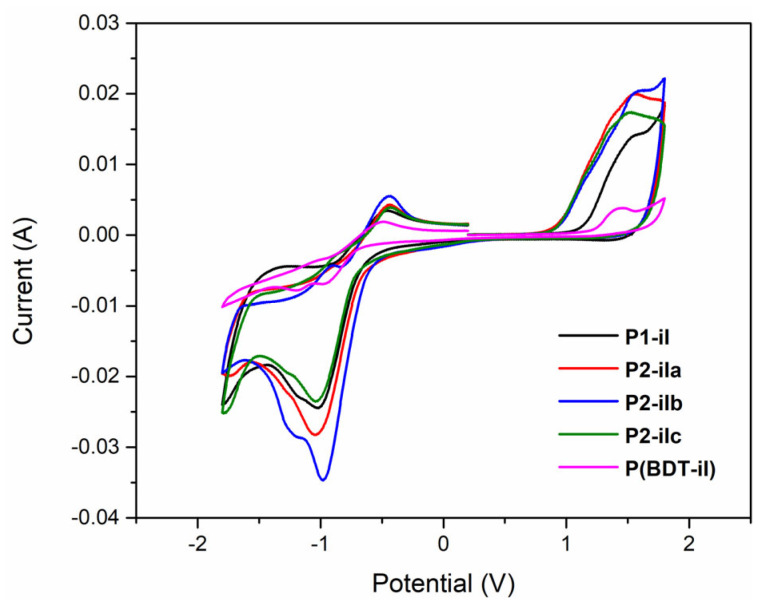
Cyclic voltammograms (second scan) of **P1-iI**, **P2-iIa-c** series, and **P(BDT-iI)** drop-cast onto glassy carbon disk electrode. Scan rate = 100 m·Vs^−1^.

CV measurements (Figure 3 and Table 2) reveal that the HOMO energy levels of terpolymers were between −5.29 and −5.31 eV for the **P2-iIa-c** series, which are shifted to higher energies compared to **P1-iI** (−5.51 eV) and **P(BDT-iI)** (−5.59 eV). These values are similar to the FOE unit (−5.28 eV), suggesting that the increased contribution of the BDT-BTzC6FOE segment produces a slight modification of the electronic distribution along the backbone. In contrast, the LUMO energy levels of the **P2-iIa-c** terpolymers (−3.66 eV to −3.77 eV) are comparable to those of **P1-iI** (−3.69 eV) and **P(BDT-iI)** (−3.67 eV), indicating that the LUMO is mainly dominated by the stronger electron-acceptor iI moieties and its essentially unaffected by FOE incorporation. As a result, the electrochemical bandgaps of the **P2-iIa-c** series (1.54–1.64 eV) are slightly lower than that of **P1-iI** (1.82 eV), primarily due to the upward shift of the HOMO energy levels. Since optical and electrochemical measurements were performed under different physical conditions (dilute solution and solid-state films, respectively), the corresponding bandgaps are not directly compared. It is worth noting that differences between optical and electrochemical bandgaps are commonly observed in conjugated polymers and have been associated with factors such as molecular organization, solid-state packing effects, exciton binding energies, and relaxation processes of photoexcited states [60,61]. Therefore, the optical and electrochemical data are used here as complementary probes of the electronic structure of the materials. Overall, these results indicate that FOE incorporation introduces only minor modifications in the HOMO energy levels and does not significantly affect the LUMO energies, indicating that the frontier orbital structure of the conjugated backbones is largely preserved. In this context, the role of FOE is better understood in terms of modulating exciton dynamics, likely through intramolecular energy transfer processes, and influencing segmental distribution, rather than significantly altering the electronic structure of the systems.

### 3.3. Photovoltaic Characterization

To investigate whether the structural and photophysical differences observed between **P1-iI** and FOE-based terpolymers translate into measurable photocurrent generation, photovoltaic parameters were evaluated using a bilayer device architecture: ITO|MoO_x_|**P1-iI**/**P2-iIb**|C_60_|Bphen|Liq|Al. This design was chosen to minimize the morphological and phase-separation effects common in BHJ systems, allowing for more direct comparisons of excitonic and interfacial charge-transfer processes. It is worth noting that bilayer configurations typically yield modest power conversion efficiencies (PCEs) due to limited interfacial area and short exciton diffusion length. In addition, electrochemical data indicate that the LUMO energy levels of terpolymers are very similar, resulting in a relatively small LUMO offset with respect to C_60_. Such a low energetic driving force may limit the efficient exciton dissociation at the polymer/fullerene interface. Therefore, rather than focusing on device performance, we emphasize relative differences in photocurrent generation between **P1-iI** and **P2-iIb** as a probe of excitonic and structural effects associated with FOE incorporation. **P2-iIb** was chosen as a representative material of the **P2-iIa-c** series since the three terpolymers exhibited similar optical and electrochemical properties.

**Figure 4:**
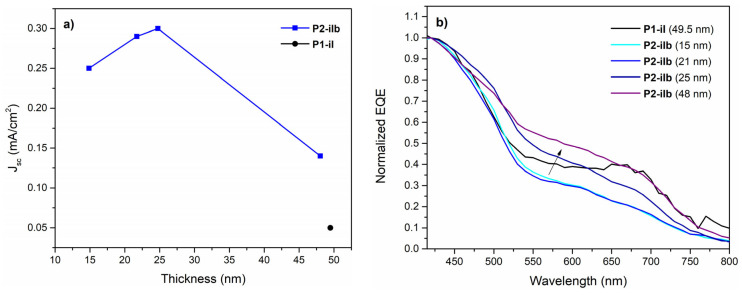
(**a**) Short-circuit current density dependence on **P1-iI** and **P2-iIb** terpolymers thickness for: ITO|MoO_x_|**P1-iI**/**P2-iIb**(x)|C_60_|Bphen|Liq|Al. (**b**) Normalized EQE spectra of bilayer PSC devices based on **P1-iI** and **P2-iIb**. The arrow highlights the spectral region where the relative EQE response increases with increasing active-layer thickness.

The photovoltaic response of **P1-iI** and **P2-iIb** was first analyzed through short-circuit current density (*J_sc_*) measurements (Figure 4). For **P1-iI**, a film thickness of 49.5 nm yielded a *J_sc_* value of 0.05 mA·cm^−2^, indicating limited photocurrent generation under bilayer conditions, as expected. In contrast, **P2-iIb** showed increased photocurrents across the range of thicknesses studied. The *J_sc_* values obtained for **P2-iIb** were 0.25, 0.29, 0.30, and 0.14 mA·cm^−2^ for active layer thicknesses of 15, 21, 25, and 48 nm, respectively. The increase in photocurrent suggests an improved balance between light absorption and exciton diffusion toward the D-A interface. The decrease at 48 nm is consistent with higher recombination losses, observed in bilayer systems with thicknesses beyond their limit exciton diffusion length.

Importantly, comparison of devices with similar active-layer thicknesses (49.5 nm for **P1-iI** and 48 nm for **P2-iIb**) revealed a higher photocurrent for the FOE-containing terpolymer. CV measurements showed that both materials had very similar LUMO energy levels and a relatively small difference in HOMO energy levels, suggesting that energetic alignment at the D-A interface is not significantly altered by FOE incorporation. Therefore, the enhanced photocurrent in the FOE-containing system is more reasonably attributed to differences in exciton generation, migration, and relaxation processes, together with possible variations in backbone organization and molecular packing induced by FOE incorporation. Although the present results are consistent with a contribution from intramolecular energy transfer processes, additional morphological effects cannot be completely excluded.

To understand the source of the enhanced photocurrent, external quantum efficiency (EQE) spectra were recorded for both terpolymers at the different film thicknesses (Figure 4). The EQE spectra were normalized at 420 nm to facilitate comparison of relative spectral contributions. Compared to **P1-iI**, an increased relative contribution in the 520–620 nm region was observed for **P2-iIb** at higher film thickness. The relative enhancement is consistent with additional population of backbone excited states, which may arise from intramolecular FRET from the FOE side groups. However, differences in film organization, thickness-dependent optical absorption, backbone organization, and charge collection efficiency may also contribute to the observed behavior and cannot be completely neglected.

Taken all together, the results presented throughout this study suggest that FOE incorporation causes measurable changes in photocurrent generation under bilayer conditions. This behavior is consistent with the dual role of FOE units identified in this study: (i) increasing backbone rigidity and altering the composition of the terpolymer during chain growth, and (ii) modulating exciton dynamics through additional intramolecular coupling pathways. The observed photocurrent enhancement likely results from the combined contribution of these structural and photophysical effects. Although overall device efficiencies were low, the systematic increase in photocurrent for the **P2-iIb** terpolymer supports the idea that FOE acts primarily as a structural and excitonic regulator rather than as a direct electronic-level modulator.

## 4. Conclusions

In this work, we systematically investigated the effect of fluorescein octyl ester (FOE) side groups on the structural, photophysical, electrochemical, and photovoltaic properties of benzodithiophene-benzotriazole-isoindigo terpolymers (**P2-iIa-c**). By synthesizing a terpolymer without FOE (**P1-iI**) and a reference **P(BDT-iI)** copolymer, we were able to identify the individual contributions of the different donor–acceptor segments along the conjugated backbone. FOE incorporation is associated with an increase in glass transition temperature, suggesting restricted segmental mobility and increased conformational constraints. Furthermore, the UV-vis results are consistent with differences in monomer reactivity during polymerization, indicating possible variations in the relative contribution of BDT-BTzFOE and BDT-iI segments within the terpolymer backbone.

These structural changes are reflected in the photophysical behavior, where the combined steady-state and time-resolved fluorescence results are consistent with the occurrence of the intramolecular energy transfer process (FRET-like) from FOE-functionalized segments toward lower-energy BDT-iI segments. This exciton redistribution leads to emission predominantly from the lowest-energy segment; however, due to the intrinsically low fluorescence efficiency of isoindigo-based units, this process does not result in enhanced radiative recombination. Despite these modifications, the electronic structure remains largely preserved, as confirmed by cyclic voltammetry. While a slight upward shift of the HOMO is observed in FOE-containing materials, consistent with the increased compositional contribution of BDT-BTzC6FOE segments, the LUMO energies remain essentially unchanged and are regulated by iI acceptor units. Under bilayer device conditions, the FOE-containing terpolymer **P2-iIb** exhibited an enhanced photocurrent compared to **P1-iI**. Given the minimal differences in frontier orbital energies, this improvement is more reasonably attributed to changes in exciton generation, migration, and relaxation processes, as well as possible differences in backbone organization, rather than variations in energetic alignment at the D-A interface.

Overall, FOE incorporation enables the modulation of segmental distribution and excitonic dynamics while preserving the electronic structure of the conjugated backbone. These findings highlight the potential of side chain engineering as a rational strategy to tune photophysical behavior in conjugated polymers without substantially perturbing their intrinsic electronic structure, which may be relevant for the design of advanced photovoltaic materials.

## Figures and Tables

**Table 1:** Number-average molecular weight, dispersity values and thermal properties of terpolymers.

Entry	*M_n_* (kDa)	Đ ^1^	TDT_10%_ (°C) ^2^	T_g_ (°C) ^3^
P1-iI	48.3	1.33	362	126
P2-iIa	14.3	1.08	376	200
P2-iIb	13.9	1.82	367	199
P2-iIc	15.7	1.08	365	216
P(BDT-iI)	7.4	1.48	366	147

^1^ Dispersity (Đ = *Mw*/*Mn*). ^2^ Temperature at 10% weight loss. ^3^ Glass transition temperature (second heating run).

**Table 2:** The optical and electrochemical properties of terpolymers **P1-iI** and **P2-iIa-c**, the copolymer **P(BDT-iI)** and the FOE antenna.

Entry	λ_max_^sol^ (nm) ^1^	λ_max_^em^ (nm) ^2^	*E_g_*^opt^ sol (eV) ^3^	$E_{on}^{ox}$ (eV)	*E_HOMO_* (eV) ^4^	$E_{on}^{red}$ (eV)	*E_LUMO_* (eV) ^4^	*E_g_*^elec^ sol (eV)
P1-iI	527	567	1.66	1.14	−5.51	−0.68	−3.69	1.82
P2-iIa	463	574	1.65	0.92	−5.29	−0.66	−3.71	1.58
P2-iIb	463	570	1.65	0.94	−5.31	−0.60	−3.77	1.54
P2-iIc	465	573	1.65	0.93	−5.30	−0.71	−3.66	1.64
P(BDT-iI)	684	812	1.63	1.22	−5.59	−0.70	−3.67	1.92
FOE ^5^	466	556	2.37	0.88	−5.28	−0.40	−4.00	1.28

^1^ Absorption maximum taken from UV-Vis spectra of the material in dilute chloroform solutions. ^2^ Emission maximum from fluorescence spectra. ^3^ Calculated from the absorption edge of the material in solution: *E_g_^opt^* = 1240·λedge^−1^ eV. ^4^ *E_HOMO_* = −e(*E_on_^ox^* + 4.4) eV/*E_LUMO_* = −e(*E_on_^red^* + 4.4) eV. ^5^ The data were taken from Ref. [25].

**Table 3:** The fluorescence lifetime for **P1-iI** and **P2-iIa-c** terpolymers, the copolymer **P(BDT-iI)** and the FOE antenna.

Entry	λ_exc_/λ_em_ (nm)	τ_1_ (A_1_) (ns)	τ_2_ (A_2_) (ns)	τ_3_ (A_3_) (ns)
P1-iI	527/567	1.10 (93%)	6.2 (7%)	-
P2-iIa	463/574	0.93 (93%)	3.1 (7%)	-
P2-iIb	463/570	0.98 (62%)	2.8 (38%)	-
P2-iIc	496/571	0.89 (88%)	2.0 (12%)	-
P(BDT-iI)	450/812	0.15 (2%)	1.4 (96%)	4.9 (2%)
FOE ^1^	458/558	2.64 (100%)	-	-

^1^ Data were taken from Ref. [25].

## Data Availability

The original contributions presented in this study are included in the article/Supplementary Material. Further inquiries can be directed to the corresponding author.

## Supplementary Materials

The following supporting information can be downloaded at: https://www.mdpi.com/article/10.3390/polym18121554/s1, Scheme S1. Synthetic scheme for terpolymers **P1-iI** and **P2-iIa-c**. Figure S1. ^1^H NMR spectrum of terpolymer **P1-iI** in CDCl_3_. Figure S2. ^1^H NMR spectrum of terpolymer **P2-iIa** in CDCl_3_. Figure S3. ^1^H NMR spectrum of terpolymer **P2-iIb** in CDCl_3_. Figure S4. ^1^H NMR spectrum of terpolymer **P2-iIc** in CDCl_3_. Figure S5. ^1^H NMR spectrum of copolymer **P(BDT-iI)** in CDCl_3_.
