# Supplementary material for: Modulating Exciton Dynamics Through Fluorescent Side Group Incorporation in Benzodithiophene-Benzotriazole-Isoindigo Terpolymers
Source: Polymers (Basel). 2026 Jun 22;18(12):1554. doi: 10.3390/polym18121554 (PMC13306971; doi:10.3390/polym18121554)
Supplement: Supplementary file 1 [file polymers-18-01554-s001.zip › polymers-4368683-supplementary.pdf]

polymers

Article

Modulating Exciton Dynamics Through Fluorescent Side Group Incorporation in Benzodithiophene-Benzotriazole-Isoindigo Terpolymers

René Hauyón [1], Yasmín Pérez [2], Daniela Zúñiga [2], Scarlet Araya [2], Bastian Camacho [2], Pablo Thomas [2], Cesar Saldías [1,3], Denis Fuentealba [1], Claudio A. Terraza [1,3], Felipe A. Angel [1,3,4] and Ignacio A. Jessop [2,*]

1 Facultad de Química y de Farmacia, Pontificia Universidad Católica de Chile, Santiago 7820436, Chile; rahauyon@uc.cl (R.H.); casaldia@uc.cl (C.S.); dlfuente@uc.cl (D.F.); cterraza@uc.cl (C.A.T.); faangel@uc.cl (F.A.A.)
2 Organic and Polymeric Materials Research Laboratory, Facultad de Ciencias, Universidad de Tarapacá, Arica 1000007, Chile; yasmin.perezmorales@gmail.com (Y.P.); z.candia.daniela@gmail.com (D.Z.); arayascarlett@gmail.com (S.A.); b.camachoromo@gmail.com (B.C.); pithomas@academicos.uta.cl (P.T.)
3 Centro de Energía, CE-UC, Pontificia Universidad Católica de Chile, Santiago 7820436, Chile
4 Centro de Nanotecnología y Materiales Avanzados, CIEN-UC, Pontificia Universidad Católica de Chile, Santiago 7820436, Chile
* Correspondence: iajessop@academicos.uta.cl or iajessop@uta.cl

Synthesis of monomers and terpolymers

Monomer 2,6-bis(trimethyltin)-4,8-bis-ethylhexyloxy-benzo[1,2-*b*:4,5-*b'*]dithiophene (1D-BDT) was obtained commercially, while the following compounds were prepared according to previously reported methods:

2-(6-bromohexyl)-4,7-bis(5-bromothiophen-2-yl)-2*H*-benzo[*d*][1,2,3]triazole (BTzC6) [1]: ^{1}H NMR (400 MHz, $CDCl_3$) δ 7.77 (d, J = 3.9 Hz, 2H), 7.48 (s, 2H), 7.12 (d, J = 3.9 Hz, 2H), 4.79 (t, J = 7.2 Hz, 2H), 2.24 – 2.12 (m, 2H), 1.46 – 1.30 (m, 6H), 0.90 (t, J = 7.2 Hz, 3H); ^{13}C NMR (101 MHz, $CDCl_3$) δ 141.85, 141.38, 131.02, 127.09, 123.14, 122.34, 113.31, 57.08, 31.34, 30.16, 26.41, 22.62, 14.13;

Octyl 2-(3-(6-(4,7-bis(5-bromothiophen-2-yl)-2*H*-benzo[*d*][1,2,3]triazol-2-yl)hexyloxy)-6-oxo-6*H*-xanthen-9-yl)benzoate (BTzC6FOE) [1]: ^{1}H NMR (400 MHz, $CDCl_3$) δ 8.98 (s, 1H), 8.25 (d, J = 7.7 Hz, 1H), 7.69 (dd, J = 11.8, 7.4 Hz, 2H), 7.29 (d, J = 7.2 Hz, 1H), 6.98 (d, J = 9.2 Hz, 2H), 6.89 (s, 2H), 6.82 (d, J = 9.1 Hz, 2H), 3.92 (t, J = 6.5 Hz, 2H), 1.30 – 1.19 (m, 4H), 1.13 (s, 6H), 1.03 – 0.94 (m, 2H), 0.82 (t, J = 7.0 Hz, 3H); ^{13}C NMR (101 MHz, $CDCl_3$) δ 175.80, 165.51, 157.99, 156.19, 134.17, 132.54, 131.36, 130.75, 130.71, 130.38, 129.92, 122.15, 114.89, 103.83, 65.93, 31.81, 29.17, 29.10, 28.27, 25.88, 22.68, 14.18 .

6,6′-dibromodi(N,N′-2-octyldodecyl)isoindigo (iI) [2]: ^{1}H NMR (400 MHz, $CDCl_3$) δ 9.05 (d, J = 8.6 Hz, 1H), 7.14 (dd, J = 8.6, 1.6 Hz, 1H), 6.86 (d, J = 1.5 Hz, 1H), 3.58 (d, J = 7.4 Hz, 2H), 1.86 (s, 1H), 1.42 – 1.17 (m, 32H), 0.87 (td, J = 6.8, 4.1 Hz, 6H); ^{13}C NMR (101 MHz, CDCl3) δ 168.22, 146.34, 132.68, 131.19, 126.80, 125.23, 120.53, 111.66, 44.83, 36.23, 32.71, 32.06, 32.01, 31.65, 30.13, 29.93, 29.79, 29.77, 29.74, 29.69, 29.49, 29.44, 26.70, 26.51, 22.83, 22.81, 14.26, 14.25.

Terpolymers **P1-iI** and **P2-iIa-c** and copolymer **P(BDT-iI)** were synthesized by Stille polycondensation using $Pd_2(dba)_3/P(o\text{-}tol)_3$ as a catalytic system at 110 °C in toluene [1,3].

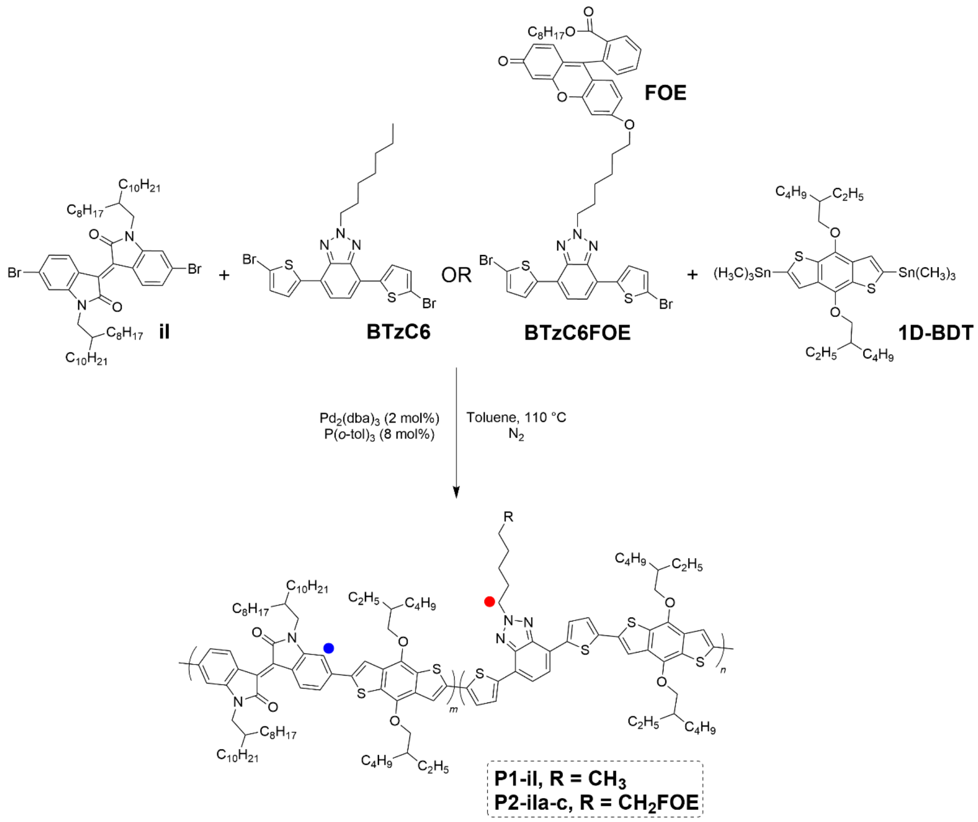

Scheme S1. Synthetic scheme for terpolymers **P1-iI** and **P2-iIa-c**.

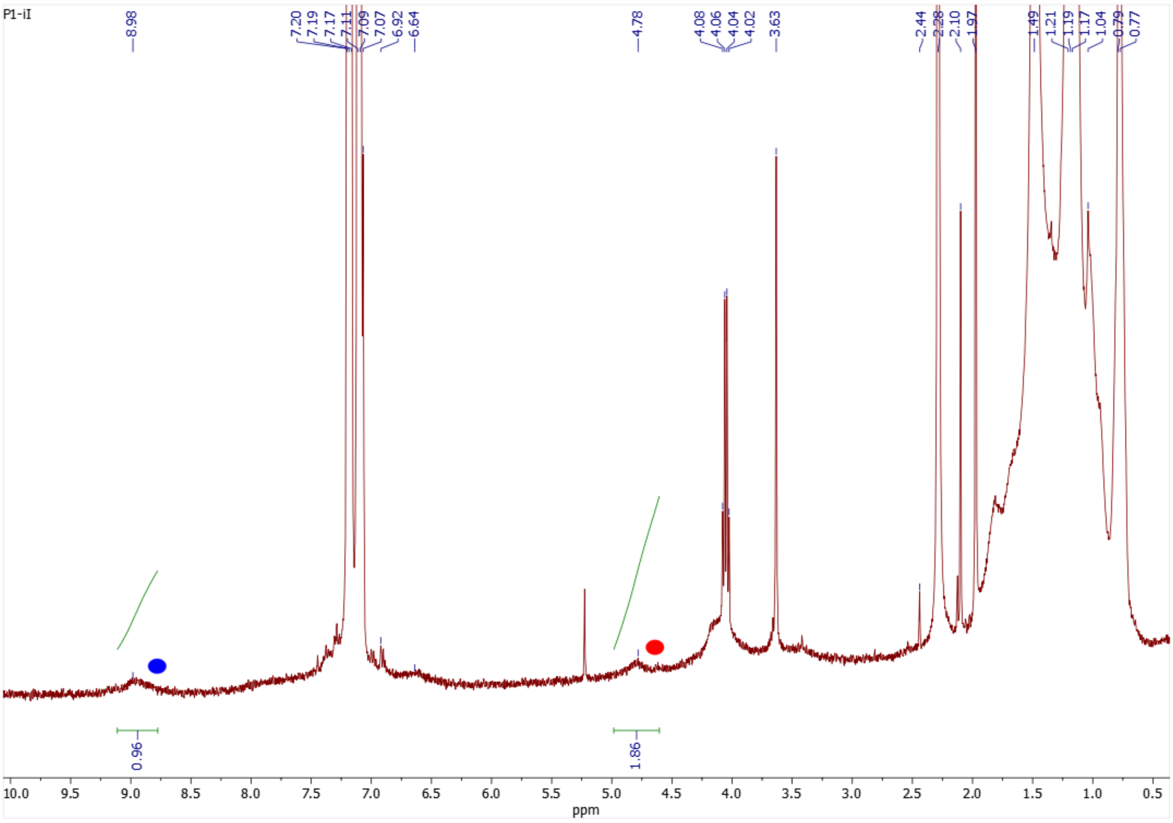

Figure S1. ^{1}H NMR spectrum of terpolymer **P1-iI** in $CDCl_3$.

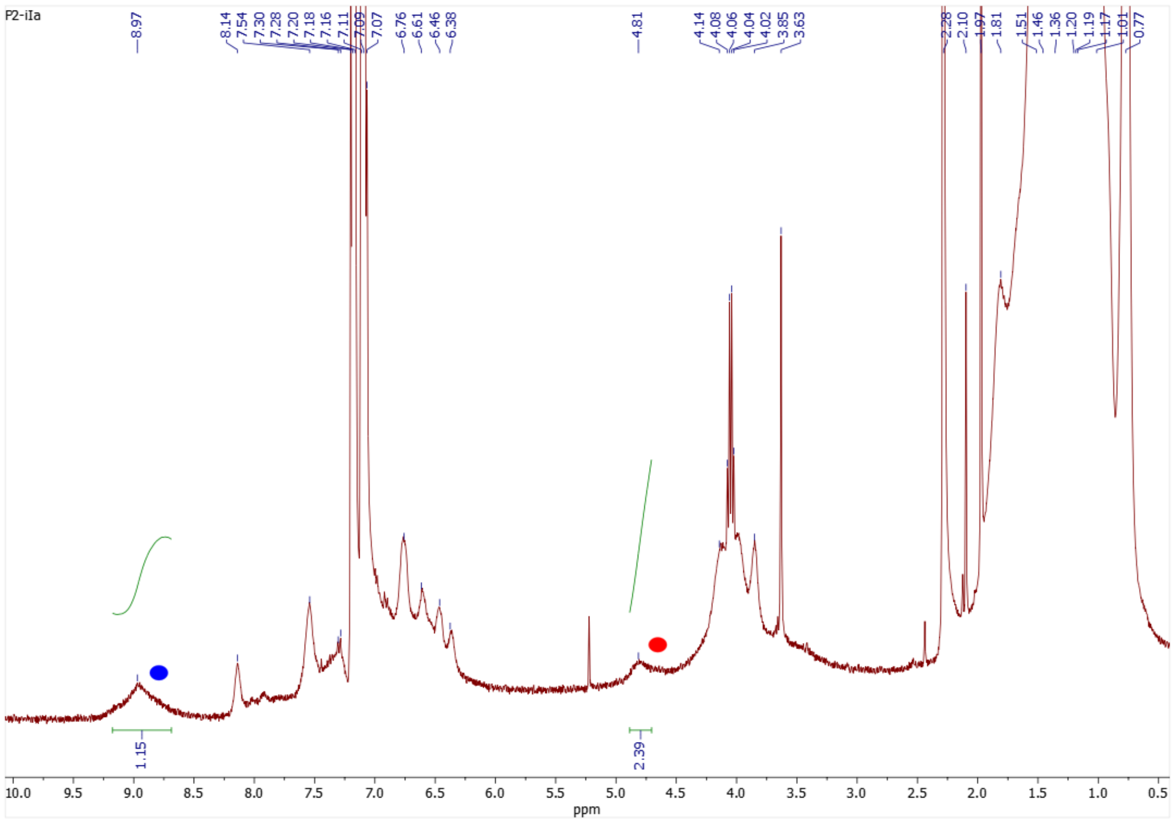

Figure S2. ^{1}H NMR spectrum of terpolymer **P2-iIa** in $CDCl_3$.

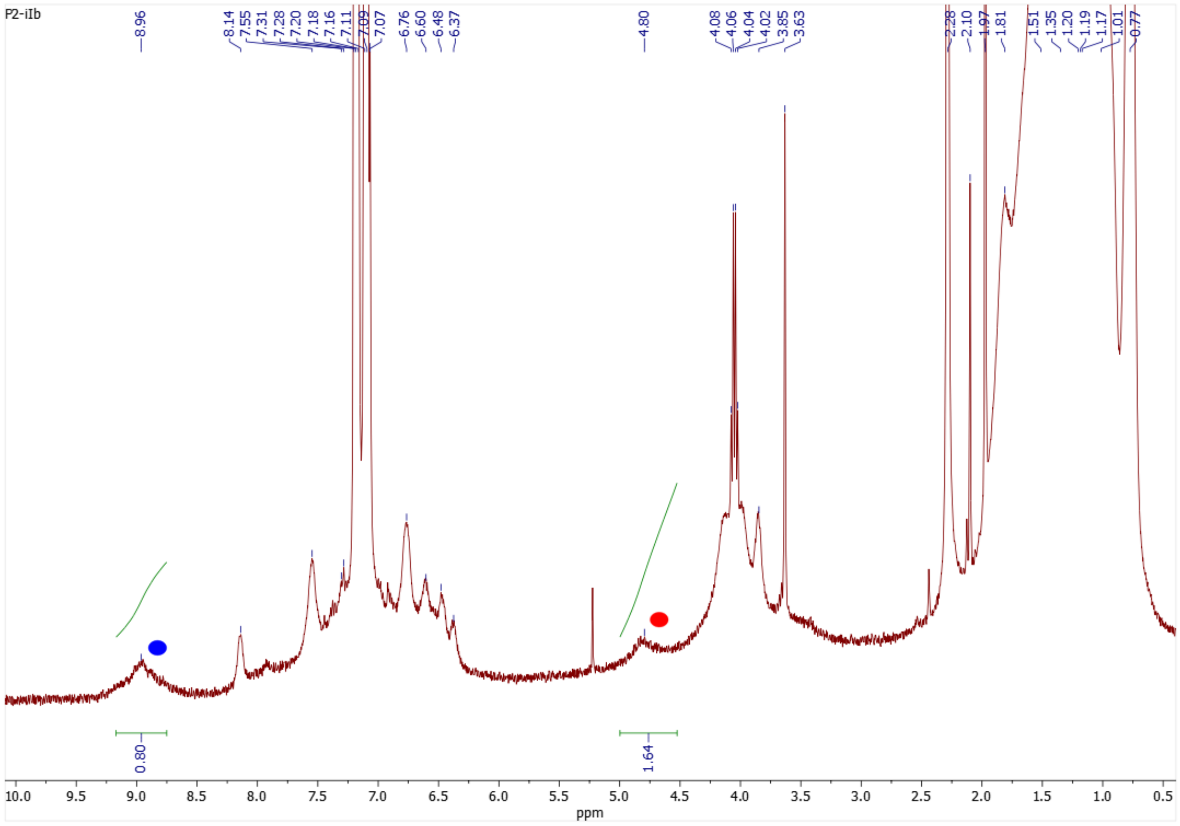

Figure S3. ^{1}H NMR spectrum of terpolymer **P2-iIb** in $CDCl_3$.

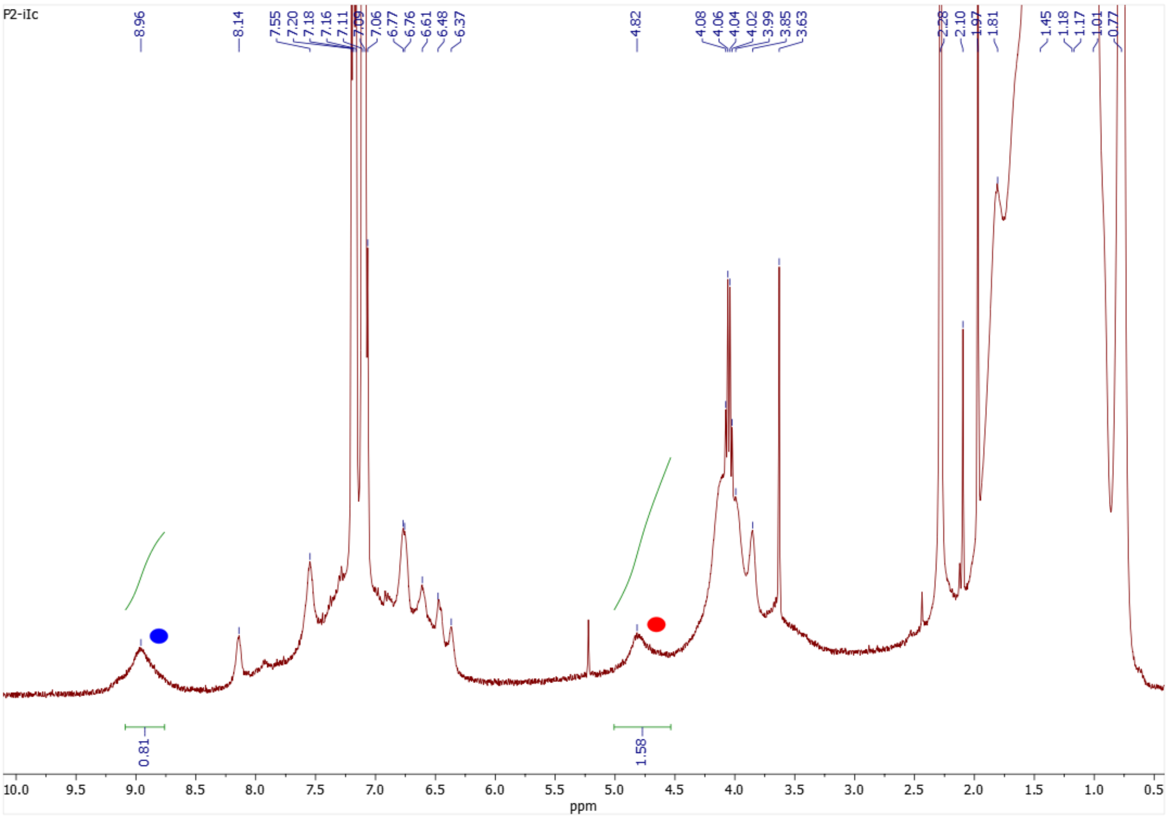

Figure S4. ^{1}H NMR spectrum of terpolymer **P2-iIc** in $CDCl_3$.

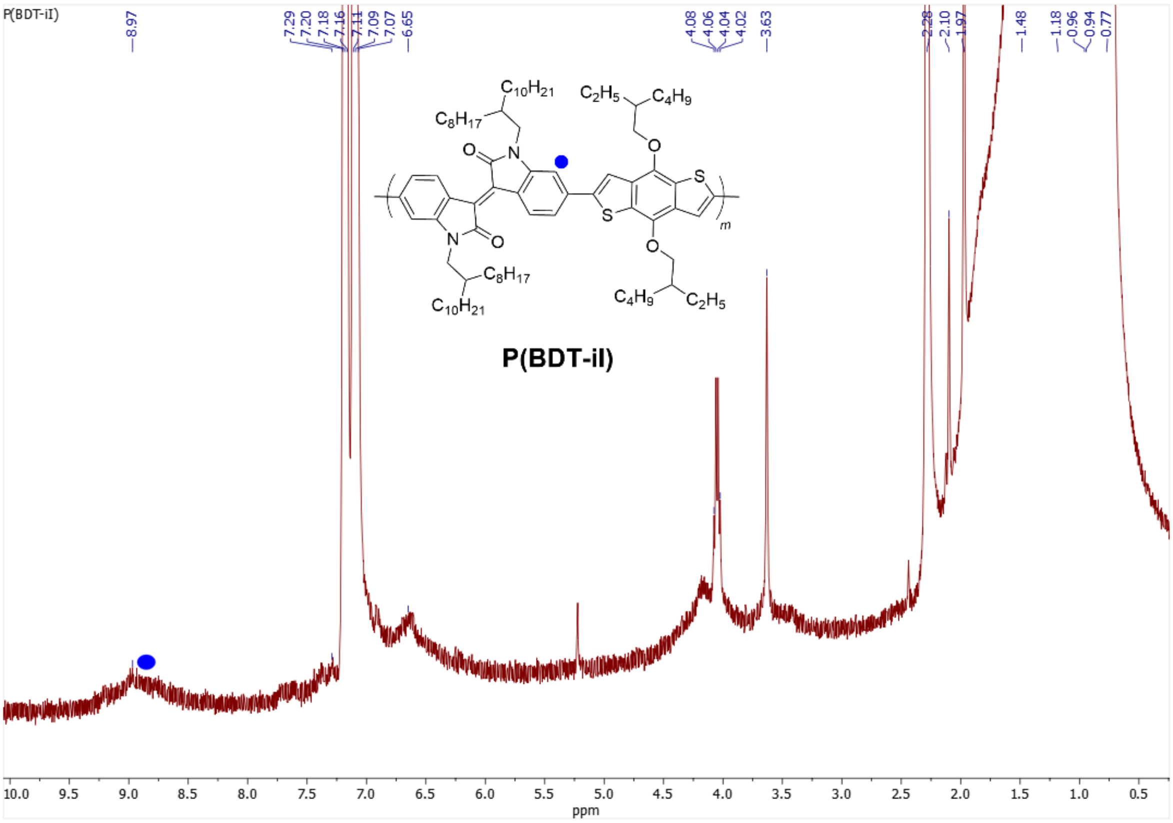

Figure S5. ^{1}H NMR spectrum of copolymer **P(BDT-iI)** in $CDCl_3$.

References

1. Jessop, I.A.; Cutipa, J.; Perez, Y.; Saldias, C.; Fuentealba, D.; Tundidor-Camba, A.; Terraza, C.A.; Camarada, M.B.; Angel, F.A. New Benzotriazole and Benzodithiophene-Based Conjugated Terpolymer Bearing a Fluorescein Derivative as Side-Group: In-Ternal Forster Resonance Energy Transfer to Improve Organic Solar Cells. Int J Mol Sci **2022**, 23, doi:10.3390/ijms232112901.
2. Grenier, F.; Berrouard, P.; Pouliot, J.-R.; Tseng, H.-R.; Heeger, A.J.; Leclerc, M. Synthesis of new n-type isoindigo copolymers. Polymer Chemistry **2013**, 4, 1836-1841, doi:10.1039/C2PY20986A.
3. Ma, Z.; Wang, E.; Jarvid, M.E.; Henriksson, P.; Inganäs, O.; Zhang, F.; Andersson, M.R. Synthesis and characterization of benzodithiophene–isoindigo polymers for solar cells. J. Mater. Chem. **2012**, 22, 2306-2314, doi:10.1039/c1jm14940g.
